# Post-Brachytherapy Transformation of Prostatic Adenocarcinoma to Squamous Cell Carcinoma: A Cadaver Anatomy Case Report

**DOI:** 10.7759/cureus.6184

**Published:** 2019-11-18

**Authors:** John T D'Angelo, Neha Bhaskar, Cole Friedes, Mujtaba Husain

**Affiliations:** 1 Medicine, University of Central Florida College of Medicine , Orlando, USA; 2 Medicine, University of Central Florida College of Medicine, Orlando, USA; 3 Pathology, University of Central Florida College of Medicine, Orlando, USA

**Keywords:** prostate squamous cell carcinoma, prostate adenocarcinoma, brachytherapy

## Abstract

Prostatic squamous cell carcinoma comprises less than 1% of reported prostate cancers. This form of cancer has a poor prognosis with a low survival time following diagnosis and no definitive form of treatment. A Caucasian male cadaver presented with primary pure squamous cell carcinoma of the prostate with metastasis to the liver and local invasion of the bladder, ureter, and rectum. Post-mortem examination showed evidence of brachytherapy radiation seeds in the prostate. Histological analysis and clinical intuition suggest that these seeds were used in an attempt to treat a previous low-grade adenocarcinoma of the prostate. The lack of patient history complicates determining a definitive cause of death, but the pathological presentation strongly suggests that the cause of death was a pure squamous cell carcinoma of the prostate following brachytherapy treatment for a previous prostatic adenocarcinoma. This case report further aids in establishing the relationship between the use of brachytherapy for prostatic adenocarcinoma and the subsequent development of prostatic squamous cell carcinoma.

## Introduction

Prostate cancer is the most common non-skin cancer affecting males, specifically with adenocarcinoma comprising up to 95% of cases [1]. In contrast, squamous cell carcinoma (SCC) of the prostate is particularly rare, as it makes up only 0.5%-1% of prostate cancers [1]. Prostatic SCC (pSCC) can present as either pure SCC or adenosquamous carcinoma [1,2]. By nature, pSCC is aggressive, often metastasizing early and forecasting a poor prognosis for patients with a median survival time of roughly 14 months [3]. Treatment of pSCC usually involves external beam radiation (EBR), chemotherapy, hormone therapy, and surgical interventions; however, these specific treatments have been ineffective in terms of providing a sustained, long-term cure [4]. 

Due to its exceeding rarity, the underlying etiology of SCC of the prostate remains largely unknown. One proposed mechanism is that pluripotent stem cells give rise to squamous cell differentiation of the prostatic urothelium, but other case reports and literature reviews have suggested that pure SCC may develop after definitive radiotherapy or hormonal therapy for low-risk prostatic adenocarcinoma [5,6]. The most common reported sites of pSCC metastasis include the liver, bone, and lungs; however, there is a limited number of studies on the extension and metastasis of pSCC into extraprostatic and intra-abdominal tissues [7]. Herein is a cadaver anatomy case report analyzing suspected post-brachytherapy transformation of adenocarcinoma to pSCC with metastasis to the liver and local extension into the bladder, ureters, and rectum.

## Case presentation

A nonagenarian Caucasian male cadaver was presented to the University of Central Florida College of Medicine Cadaver Laboratory following post-embalming processes. The authors could not ascertain the patient's clinical history through medical records, and details from the death certificate were lacking. The pathological presentation at the time of dissection strongly suggests that the cause of death was pure SCC of the prostate.

**Figure 1:**
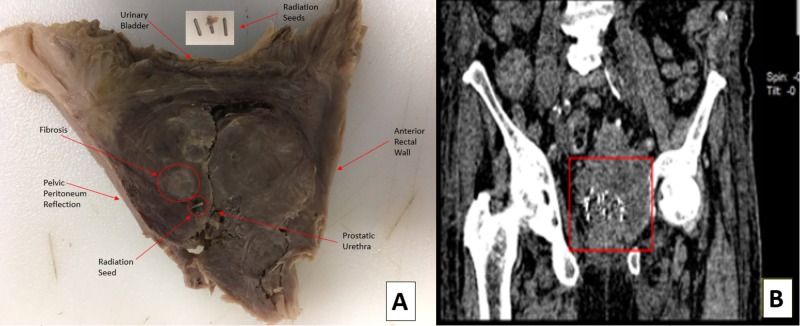
(A) Prostate and surrounding tissue specimen with radiation seeds noted. (B) Radiation seeds within the prostate depicted in a post-mortem CT.

Careful examination of the skin did not reveal any obvious deformities or abnormalities including radiation tattoos or skin injury. Full body computed tomography (CT) imaging showed numerous metallic bodies in an enlarged prostate gland (Figure 1A, 1B). Anatomic dissection of the abdominal and pelvic regions demonstrated prostatic enlargement, palpable bladder hardening, and bilateral hydroureters.

**Figure 2:**
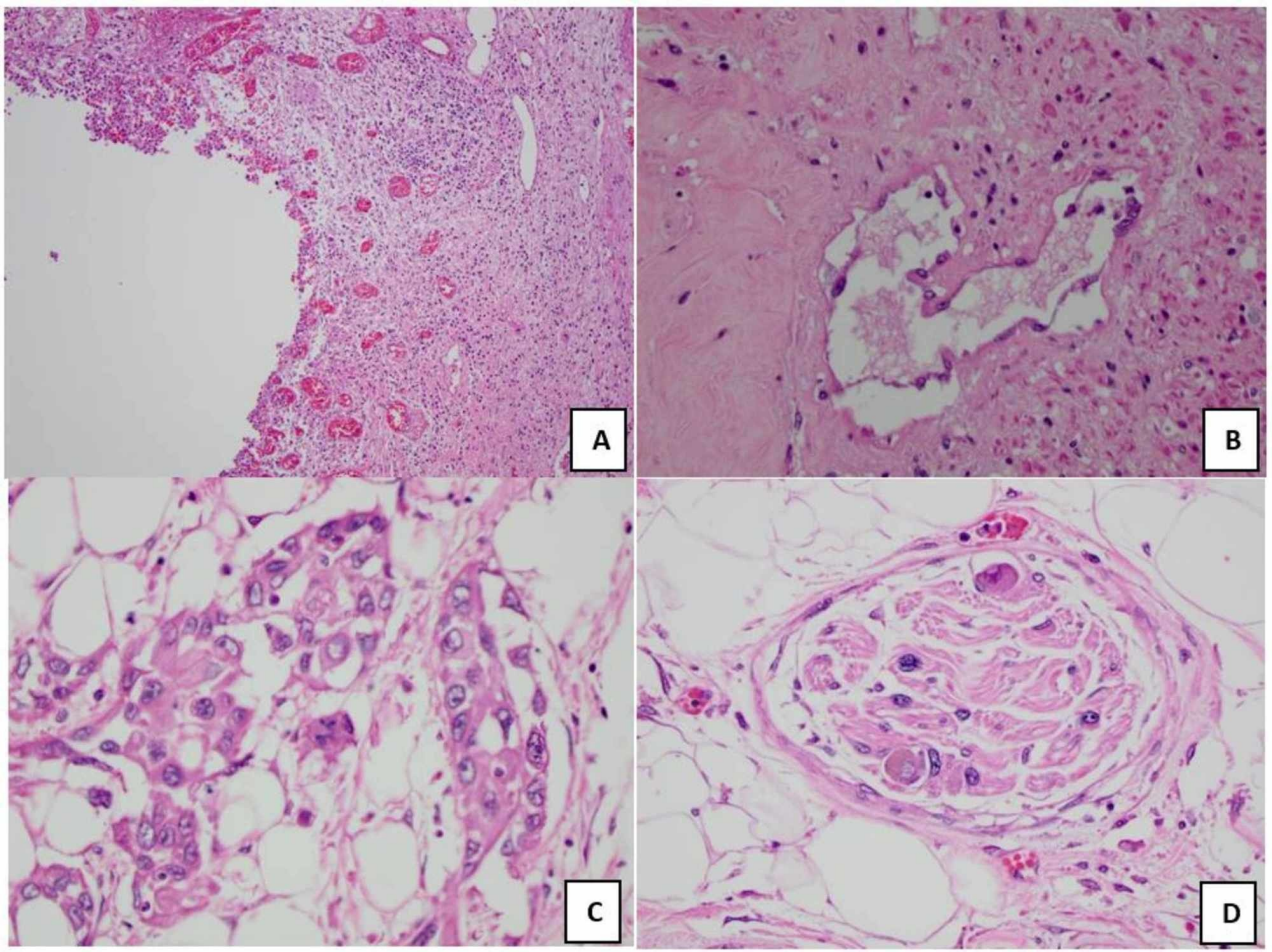
(A) Prostatic urethra with necrotic tissue and an ulcerated epithelium. (B) Prostatic stroma with fibrosis and no adenocarcinoma. (C) Keratinized SCC within posterior prostatic capsule. (D) Keratinized SCC within prostatic neurovascular features. SCC, squamous cell carcinoma

Histological analysis of the prostate revealed an ulcerated and necrotic prostatic urethra near the epithelial layer (Figure 2A). Prostatic stromal fibrosis was also seen on histological analysis, and there was no adenocarcinoma in ductal regions (Figure 2B). Further examination of the prostatic urethra revealed SCC along the epithelial layer with extension into the posterior prostatic lobe (Figure 2C). In addition, there was evidence of SCC within the periprostatic fat and prostatic perineural tissue (Figure 2D). However, there was no noted SCC infiltration into the prostate’s lymph nodes. The pSCC also showed signs of keratinized tissue surrounding the squamous cells. There were no features of adenocarcinoma as the remaining glandular features of the prostate had a basal cell layer and no prominent dark nucleoli in the secretory layer. No immunohistochemical (IHC) stains for prostatic adenocarcinoma were performed at the time of analysis. Therefore, the diagnosis of pSCC was made by histology alone.

**Figure 3:**
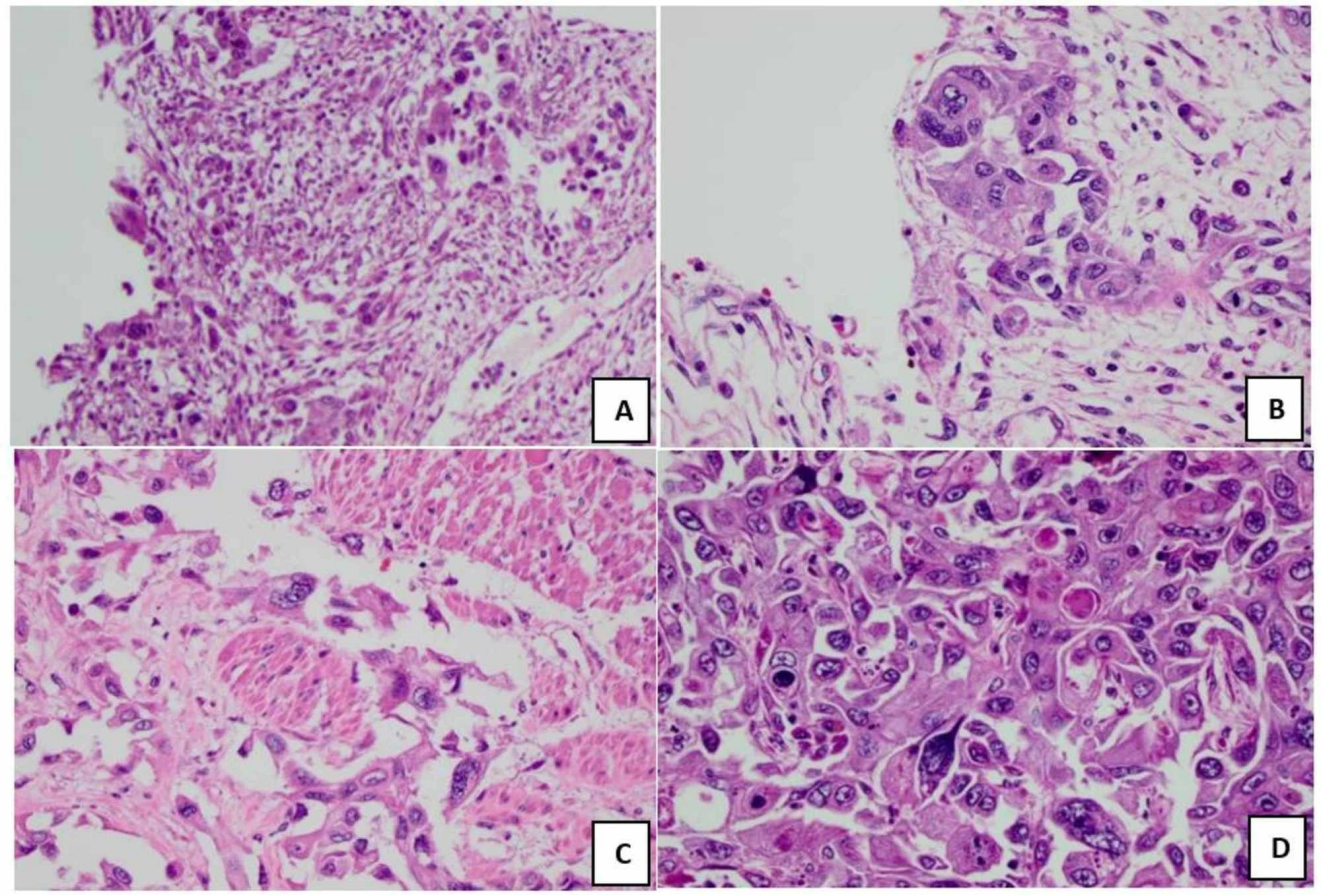
(A) SCC invasion of bladder trigonal epithelium. (B) SCC invasion of left ureter epithelium. (C) SCC invasion of rectal smooth muscle. (D) Liver’s hepatic lobules with SCC invasion. SCC, squamous cell carcinoma

**Figure 4:**
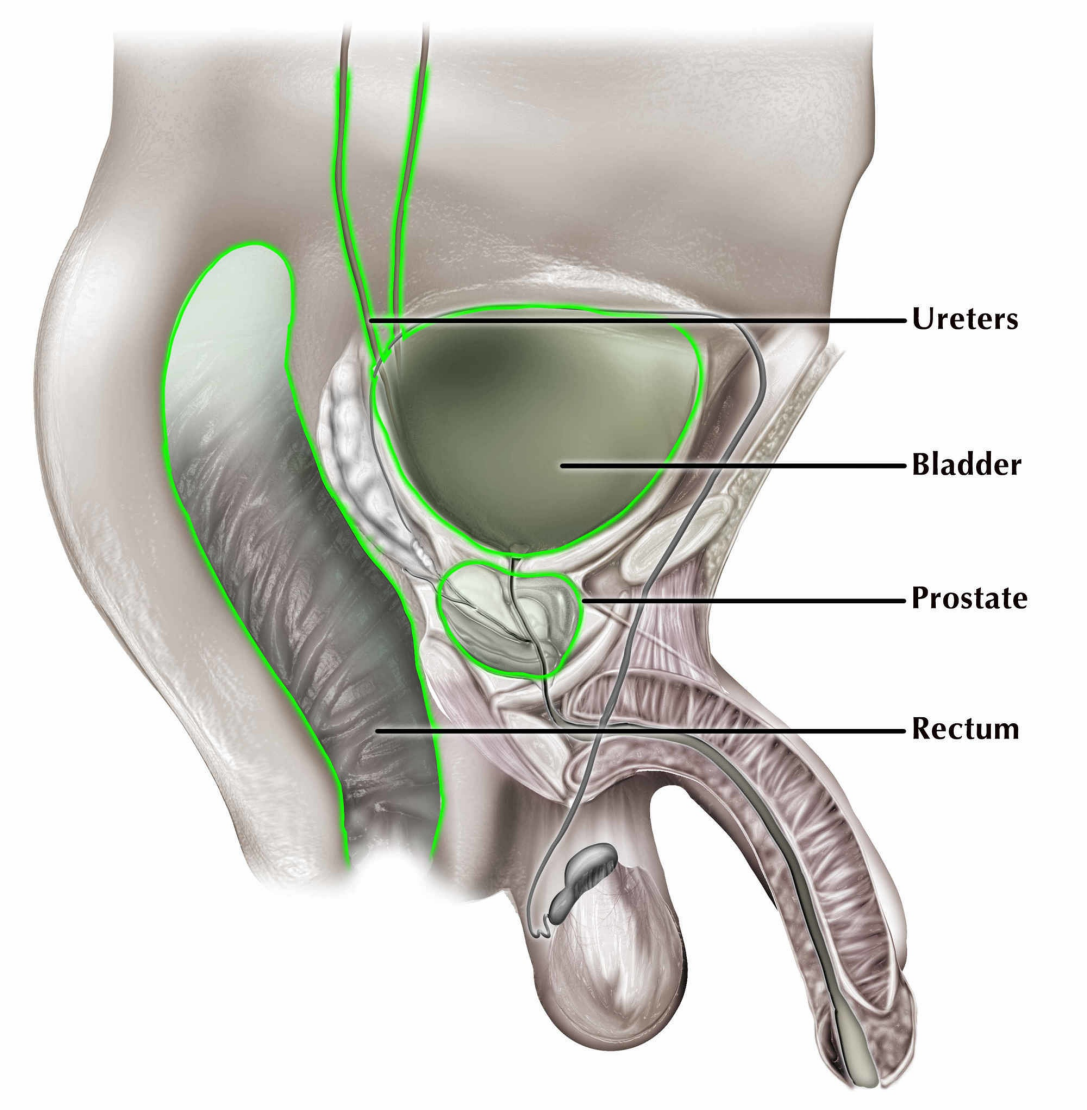
This diagram depicts the prostatic SCC's local invasion and the green outline represents locations of the SCC. SCC, squamous cell carcinoma

Once the diagnosis of pSCC was suspected, common sites of bony metastasis including the pelvis, femurs, and thoracic and lumbar spines were excluded by close examination of the CT imaging at these locations. Additional sites of possible metastasis including the bladder, rectum, and liver were then examined. Histological analysis revealed SCC invasion into the bladder trigone (Figure 3A), ureteral epithelium (Figure 3B), rectal smooth muscle (Figure 3C), and hepatic lobules (Figure 3D). These sites of metastasis also showed keratinization similar to what was observed in the prostate. In addition, there were no signs of neurovascular infiltration into any of these four sites unlike the prostate. All the sites of local SCC infiltration are illustrated in Figure 4.

## Discussion

The results provided from extensive anatomic dissection, histologic examination, and CT imaging suggest the patient’s cause of death was primary pSCC with metastasis to the liver and local infiltration of the ureters, bladder, and rectum. SCC of the bladder is more common than pSCC as it comprises 2%-5% of all bladder carcinomas, and because bladder SCC arises within the same anatomic region as pSCC, it is important to exclude it as the cause of death [8]. The rarity of pSCC has made it difficult to study and define, but Mott defined strict criteria: A) malignant neoplastic growth with disordered growth and anaplasia; B) squamous cell differentiation with keratinization and intercellular bridges; C) lack of glandular tissue within the cancerous region; D) no previous estrogen therapy; and E) absent primary squamous cancer in other regions of the body, especially in the bladder [9]. This patient clearly fits the first three criteria based on histological analysis and the following discussion provides evidence that the last two criteria apply here.

To address the final Mott criteria, the bulk of the tumor was located in the prostate as the qualitative density of SCC at the additional sites was much less compared to the prostate. More specifically, the SCC in the bladder was confined to the trigone and there was no neurovascular infiltration within the bladder. Additionally, necrotic tissue was noted in the prostate, and this key feature further points to a carcinoma of prostatic origin [10]. Altogether, this evidence supports the hypothesis that the tumor originated in the prostate and not the bladder, and this fulfills the fifth criteria proposed by Mott for primary pSCC. Mott suggested that pSCC usually causes osteolytic bone lesions, but these were not noted in this case as the primary tumor spread directly to the rectum, ureters, and bladder, and indirectly to the liver via vascular infiltration.

At the time of dissection, IHC stains were unfortunately not performed. IHC analysis was considered to help distinguish the anatomic origin of the SCC; however, it has been postulated that IHC profiles remain ineffective for diagnosing pSCC as the tumor cells lose reactivity for prostate specific antibodies secondary to squamous differentiation [4]. More specifically, stains for prostate specific antigen and prostatic acid phosphatase are negative in cases of pSCC [4]. In addition, high-molecular-weight keratin stains are positive for both pSCC and bladder SCC [4,11]. Therefore, staining for these common pSCC markers would not have further elucidated the SCC's origin, but the evidence provided from histological and gross analysis is strong as previously stated.

The prevalence of prostatic adenocarcinoma (95%) and the positive prognosis of prostatic adenocarcinoma (98% survival after five years) support the notion that the primary cancer that first affected this patient was an adenocarcinoma [12,13]. As previously stated, one of the proposed etiologies of pSCC is induction by radiation or hormonal therapy for adenocarcinoma. An accepted timeline for the development of pSCC after the use of radiation or hormonal therapy has not been established; however, reports indicate the development of a secondary pSCC ranging from 18 months to 10 years after initial primary diagnosis [2]. Therefore, it is most likely that the total intraprostatic time of brachytherapy seeds in our patient is similar, and the original diagnosis would have been made in a similar timescale. In 2012, there was evidence that high-dose and low-dose brachytherapy were being used more frequently to control low- to favorable intermediate-risk, local prostatic adenocarcinoma [14,15]. As high-dose brachytherapy seeds are only implanted temporarily, the patient most likely had low-dose brachytherapy treatment. These recommendations are similar today, with updated guidelines highlighting the importance of shared decision making when considering the use of brachytherapy for clinically localized prostate cancer [16-18]. In addition, combination therapy involving brachytherapy and hormonal therapy without external beam radiation therapy (EBRT) is not recommended for prostatic adenocarcinomas of low-, intermediate-, or high-risk groups [19]. Combination therapy involving EBRT, brachytherapy, and hormonal therapy is recommended for patients in unfavorable intermediate- and high-risk groups, but the use of EBRT can be ruled out based on the lack of external scarring on this patient’s skin [19]. Finally, brachytherapy for pSCC treatment is not a common practice [4]. All of these points enhance our suspicion that the brachytherapy was not used to treat our patient's pSCC seen post-mortem, but rather for a previous low-risk or favorable intermediate-risk adenocarcinoma. The strong likelihood of isolated brachytherapy treatment without estrogen therapy supports the fourth Mott criteria, and for these reasons, this patient most likely had a primary SCC of the prostate.

This report further supports the proposed relationship between radiation therapy for low-risk adenocarcinoma and the subsequent development of a pSCC. The literature has shown that this radiation-associated secondary malignancy carries metastatic potential, and the findings of this report further substantiate this [4]. While the rarity of pSCC has been noted, literature reviews have summarized 66 cases of pSCC that arose either de novo or after therapeutic intervention [2]. Seven out of 27 pure pSCCs arose after treatment for histologically confirmed adenocarcinoma; 50% of total pSCCs (including adenosquamous and pure SCC) developed after previous treatment in the form of either radiation or hormonal therapy [2]. The findings of our report are even more rare because there was no evidence of adenosquamous cell differentiation in our patient’s prostate, even though roughly 80% of those who develop pSCC after previous diagnosis of prostatic adenocarcinoma show squamous cells mixed with adenosquamous cells [2]. By our best estimated knowledge, this is one of the few case reports demonstrating the development of a pure pSCC without adenosquamous features following brachytherapy intervention.

## Conclusions

pSCC is a rare form of cancer that carries a poor prognosis consisting of a low survival rate and a high rate of metastasis with an indeterminate treatment course. Given its rarity, the etiology is not well understood, although the literature suggests a relationship between treatment of adenocarcinoma of the prostate and subsequent development of pSCC. While this report makes assumptions about our patient's clinical history and management, there exists value in post-mortem dissection for understanding the development of rare secondary cancers. Along these lines, the histological and gross analysis indicates a sequence of events that is currently underreported: pure pSCC following brachytherapy for a previous prostatic adenocarcinoma. The novelty of this case is furthered by the lack of adenosquamous features in this pSCC and the extraprostatic spread to the bladder, ureters, rectum, and liver. This case is presented with the intention of strengthening the relationship between prostatic brachytherapy and subsequent development of pSCC. This will contribute to the understanding of the underlying etiology of pSCC and enhance knowledge of diagnostic courses for improved long-term outcomes.
